# Supplementary material for: Mapping enzyme catalysis with metabolic biosensing
Source: Nat Commun. 2021 Nov 23;12:6803. doi: 10.1038/s41467-021-27185-9 (PMC8611028; doi:10.1038/s41467-021-27185-9)
Supplement: Supplementary file 3 — Description of Additional Supplementary Files [file 41467_2021_27185_MOESM3_ESM.pdf]

Title: Supplementary Data 1.
Description: Raw data of m/z peaks for each corresponding wells. After total ion normalization, binning and averaging the signals of all wells, 399 m/z peaks that are three times above the background are selected (the 19 m/z peaks used for UMAP clustering are included). Wells that do not have signal intensity above the background threshold (43 wells) or that are abnormally high (17 wells) are removed, resulting a total of 9940 wells that proceed in the analysis.

Title: Supplementary Data 2.
Description: Fold change of m/z peaks and adjusted significant P value for the TAL cluster and AHP cluster using a two-sided Wilcoxon Rank Sum test.

Title: Supplementary Data 3.
Description: Sequences at the 4 active site positions (199 202 259 261) of the top screened mutations of g2ps1 enzyme for TAL and AHP respectively and their bulk production levels verified by HPLC tandem MS.

Title: Supplementary Data 4.
Description: Sequences at the 4 active site positions (199 202 259 261) of mutations recovered from TAL and AHP clusters based on the UMAP analysis.

Title: Supplementary Data 5.
Description: HPLC tandem MS analysis of Library for TAL

Title: Supplementary Data 6.
Description: HPLC tandem MS analysis of Library for AHP

Title: Supplementary Data 7.
Description: Plasmid sequences of ptef-2ps.

Title: Supplementary Movie 1.
Description: Three dimensional UMAP featured with types of colonies in wells as shown in Supplementary Fig. 10b.

Title: Supplementary Movie 2.
Description: Three dimensional UMAP as shown in Supplementary Fig. 10c.
